# The Global Impact of the COVID-19 Pandemic on Individuals' and Couples' Sexuality

**DOI:** 10.3389/fpsyg.2021.798260

**Published:** 2022-01-06

**Authors:** Stefano Eleuteri, Federica Alessi, Filippo Petruccelli, Valeria Saladino

**Affiliations:** ^1^Faculty of Medicine and Psychology, Sapienza University of Rome, Rome, Italy; ^2^Institute for the Study of Psychotherapy, Rome, Italy; ^3^Department of Human and Social Sciences, University of Cassino, Cassino, Italy

**Keywords:** COVID-19, sexuality, sexual dysfunctions, sexual health, pandemia

## Abstract

The COVID-19 pandemic and its related restrictions significantly impacted individuals' health, wellbeing, and security. Isolation, limitation of movement, social distancing, and forced cohabiting have had a strong influence on all areas of people's lives as well as on their sexuality. Investigating how the COVID-19 outbreak and its consequences impacted people's sexuality was the primary aim of this review. Particularly, we focused on: (1) the variables associated with the improvement or the deterioration of individuals' and couples' lives during the pandemic; (2) the use of sex as a coping strategy; (3) the impact of COVID-19 outbreak on LGBT people. Results have shown that the worsening of sexual life seems to be related to couples' conflict, emotions and psychological difficulties, being female, being single or away from the partner, being a health care worker, and having children. Moreover, a detrimental effect on sexuality was associated with stress, forced cohabitation, routine, anxiety and worry about the job and the pandemic, feeling partner distance, being unhappy with their partner, and lack of privacy. On the other hand, improvements in sexuality were associated with living happily with a partner, being happy and satisfied with a partner, feeling less stressed and more bored, having more free time, having fewer recreation opportunities, and having minor workload. During the pandemic, there was an increase in using sex toys, pornography consumption, masturbating, and trying sexual experimentations. Among LGBT people, an increase was found in the number of casual sexual partners potentially due to the perceived lower likelihood of transmission through sex. Moreover, the increase in sexual activity may have represented a coping strategy to quarantine-related distress.

## Introduction

On March 11, 2020, the WHO Director-General declared COVID-19 (COronaVIrus Disease 19) a global pandemic (World Health Organization, 2020). All over the world, the primary aim was reducing the spread of the virus by adopting containment measures including mobility and sociality restrictions, and closure of non-essential production activities and services.

During the pandemic, people's lives dramatically changed and individuals had to cope with many stressors such as social isolation, recreative, physical, and social limitations, school closures, forced cohabitation, financial uncertainty, fear of contagion, and death. Such an emergency negatively affected individuals' psychological health worldwide, triggering stressful emotional responses characterized by an increase in negative emotions such as anxiety, depression, and a decrease in positive emotions and life satisfaction (Li S. et al., 2020; Pera, 2020; Salari et al., 2020). For instance, recent research has shown that this situation induced a change in sleep-wake rhythms and a decrease in sleep quality (Cellini et al., 2020) and an increase in anxiety, depression, and harmful alcohol abuse (Ahmed et al., 2020; Cao et al., 2020; Pera, 2020). Other common responses to the pandemic were fear, anger, sense of guilt, pain, and loss, and post-traumatic stress-related symptoms (Gawai et al., 2020; Pera, 2020).

Sexuality is a central part of human beings' lives that is influenced by several factors (WHO, n.d.). The World Health Organization (WHO, n.d.) defines sexual health as “*a state of physical, emotional, mental, and social well-being in relation to sexuality; it is not merely the absence of disease, dysfunction, or infirmity.”* To prevent the spread of the virus, some countries recommended following some suggestions, for example, avoiding sex parties, limiting the number of sexual partners, increasing virtual sexual activities, avoiding kissing, wearing a mask during sex, trying new sexual positions to prevent face-to-face contact, masturbating together keeping a moderate distance, using condoms and dental dams, and washing and disinfecting themselves and sexual toys before and after sex (Quotidiano sanità, 2020; Avert, 2021; NYC Health, 2021). In this frame, our aim was to investigate how all these pandemic-related changes are affecting people's sexual lives.

## Materials and Methods

For the present narrative review, we collected 105 scientific articles found using PubMed and PsicInfo. We used keywords such as “COVID-19,” “sexuality,” “sexual dysfunctions,” “sexual health,” “coping strategy,” “couples,” “LGBT.” The literature review was conducted from July to September 2021. After reading the full texts, we selected 19 articles for this review, specifically, 14 cross-sectional studies, 2 observational studies, a case-control study, a critical commentary, and a global analysis.

The inclusion criteria were as follows: (1) articles written in the English language; (2) articles on the COVID-19 pandemic's impact on individual's and couples' sexuality; (3) articles on the use of sex as a coping strategy during the COVID-19 pandemic; (4) articles on the COVID-19 pandemic's impact on the LGBT community; (5) articles published from 2020 to 2021; and (6) articles reporting empirical results. The exclusion criteria were as follows: (1) articles in languages other than English; (2) articles not related to the topic of interest.

## Results

### Love and Sex During the Pandemic

According to WHO (2020), sexual health constitutes individual well-being at a physical, emotional, mental, and social level while sexual dysfunctions can be seen as a condition that prevents people from experiencing satisfaction from sexual activity (Karagöz et al., 2020). Factors underlying sexual dysfunctions can be both organic (i.e., vascular, hormonal, etc.) and/or psychogenic (i.e., anxiety, depression, etc; Hedon, 2003; Clayton and Groth, 2013). All around the world during the pandemic, prolonged quarantine, social distancing, and home confinement impacted people's sexuality in different ways.

A cross-sectional study conducted in Bangladesh, India, and Nepal from April 3 to April 15, 2020, found that 45% of participants thought that quarantine affected their sexual lives, even if no substantial differences in sexual activity were found between before and during lockdown (Arafat et al., 2020). Indeed, percentages of respondents that reported engaging in sexual activity with their partner one to five times a week were 76% in the pre-pandemic period and 72% during the pandemic. Compared to the period prior to quarantine, during lockdown 3.3% of participants reported an increase in sexual activity from 1 to 5 times a week to more than 5 times. Furthermore, half of the sample (50%) reported a greater emotional bonding since quarantine started (Arafat et al., 2020).

In a survey conducted on a sample aged 18 or older living in Australia, participants were asked about their sexual lives during all of 2019 and the period after March 22, coinciding with the lockdown period (Coombe et al., 2021). Responders reported a median of one sexual partner in both time windows considered but 89.8% said they had sex in 2019 while 60.3% said they had sex during lockdown. Only 14.3% of participants affirmed that they were having more sex during lockdown vs. the remaining participants (53.5%) who suggested a decrease in sex frequency. The latter were mainly single (69.1%). Moreover, men who have sex with men (MSM) said they experienced less sexual activity during lockdown compared to 2019, while respondents who lived with their partner were more likely to report the same amount of sex or an increase in sex frequency during lockdown compared to 2019. A small percentage of participants (1.2%) said they participated in group sex, swinging, or threesomes since lockdown began, compared with 2019 (Coombe et al., 2021).

Findings also showed a change in sexual practices. Participants reported an increase in the use of sex toys on their own and masturbating during the pandemic, especially if they reported less or no sexual activity during lockdown compared with those who reported an increase in frequency. Moreover, 11.5% admitted to buying a sex toy since COVID-19 began and of these, 24.0% admitted that it was their first (Coombe et al., 2021).

COVID-related stressors may predict a decrease in relational satisfaction and an increase in maladaptive relationship behaviors, such as conflict (Balzarini et al., 2020; Luetke et al., 2020). Balzarini et al. (2020) found that participants who reported COVID-related stressors (i.e., greater loneliness, financial hardship, and stress) were more likely to find a decrease in satisfaction regarding their relationship and increased conflict, although perceiving their partner as highly responsive mitigated this association.

The 2020 National Survey of Sexual and Reproductive Health During COVID-19 (NSRHDC) conducted online on a sample of 1,010 adults between April 10 and April 20, demonstrated that coronavirus-related conflict can negatively affect intimate behaviors as well as solo and partnered sexual activity (Luetke et al., 2020). Particularly, participants who reported more coronavirus-related conflicts (34%), comparted with who did not, were likely to find a reduction of hugging, kissing, cuddling, or holding hands with their partner; a decreased frequency in solo and partnered masturbation; a reduction in giving or receiving oral sex; and a decrease in complete sexual intercourse. In addition, results reported a negative correlation between levels of conflict and frequency of sexual behaviors: as the levels of conflict increased the sexual behaviors frequency decreased. This trend was found to be stronger among men than among women. Luetke et al. (2020) supposed that this could be due to women's propension to engage in sex to protect the relationship, as shown in previous research (Traeen and Skogerbo, 2009). Moreover, among those who confirmed they had engaged in sexual activity in the last month, conflict was associated with lower levels of self-reported orgasm and emotional connection with their partner, even if in a non-significant way (Luetke et al., 2020).

Emotions and psychological difficulties related with the COVID-19 outbreak altered people's desire for intimacy and sexual satisfaction. Cocci et al. (2020) found that during quarantine levels of satisfaction decreased: 53.53% of responders reported no satisfaction during lockdown. Sexual dissatisfaction in both men and women was significantly correlated with younger age and depression levels, while sexual dissatisfaction in women was also significantly correlated with knowing someone positive with COVID. However, 40% of participants felt an increase of sexual desire during lockdown, even if this improvement did not translate into an increase of sexual activity frequency (Cocci et al., 2020).

An online survey conducted by De Rose et al. (2021) from April 8 to May 2 investigated the effect of lockdown on sexual desire, sexual satisfaction, and depressive symptoms in a sample of healthcare workers and their relatives and friends. Results showed that, despite of employment, 90% of the participants reported a reduction in the levels of sexual satisfaction predicted by depressive symptoms, low sexual desire, and living with their partner. Moreover, women working as healthcare workers, living with their partner, and having low sexual satisfaction experienced low levels of desire (De Rose et al., 2021).

The increase in household duties on women, because of the impossibility to be supported by the family, nannies, housekeepers, etc., can explain the reduction of sexual desire (Craig and Churchill, 2020; Collins et al., 2021; De Rose et al., 2021). Moreover, school closures and the cessation of extracurricular activities may have prevented women from sharing intimate moments with their partner. The findings can also be due to the stress associated with the increase in workload and responsibilities and to the greater possibility of being infected because of the close contact with COVID-19 patients. The lower desire in people living with their partner can be due by the fear of infecting them as well (De Rose et al., 2021).

In this regard, an online survey conducted on a sample of men living in the Western Black Sea Region from June 8 to June 25, 2020 revealed that individuals who worked in healthcare were slightly more likely to report the fear of COVID-19 infection during sexual intercourse compared to individuals from other professional groups (Baran and Aykac, 2021). Interestingly, health workers compared to others were more likely to experience the fear of infecting their partners. The greater possibility to be infected and the tendency to protect their loved ones may have led to maintaining distance from their partners, leaving home or living in a separated room, and, consequently, to a reduction in sexual intercourse frequency (Baran and Aykac, 2021).

The COVID-19 impact on women's sexuality was confirmed by several studies showing an increase in female sexual disfunctions (Karagöz et al., 2020; Schiavi et al., 2020; Yuksel and Ozgor, 2020). Schiavi et al. (2020) found a significant decrease in Female Sexual Function Index (FSFI) scores during the pandemic compared to the pre-pandemic period in a sample of 89 Italian reproductive-age women, aged 28–50, living with their partners. Moreover, results showed a general decrease in intercourses frequency per month. A significant worsening in sexual function and quality of life, a decrease in the mean number of sexual intercourse sessions per month, and higher levels of distress were found in women working outside the home during quarantine. For women with a higher level of education during the COVID-19 pandemic, a significant difference was found in the number of intercourse sessions per month, in the FSFI score, and in quality of life but not in distress levels. Finally, women with one or more children showed a significant difference in FSFI scores during the COVID-19 outbreak (Schiavi et al., 2020).

In a study conducted on a sample of Turkish women found a significant increase in frequency of intercourse during the pandemic and a significant decrease in the quality of sexual life compared to 6–12 months prior to the pandemic (Yuksel and Ozgor, 2020). Moreover, during the COVID-19 quarantine, women reported a lower desire to become pregnant, a lower intent in using contraception, and an increase in menstrual disorder compared to before. Furthermore, FSFI scores were better 6–12 months prior to the pandemic than during the pandemic (Yuksel and Ozgor, 2020).

During quarantine, high desire to engage in sexual activities without commitment was associated with a greater negative impact on subjective well-being for women, but not for men (Wignall et al., 2021). In a sample of 565 UK residents, aged 18–32, Wignall et al. (2021) found sexual desire to be higher for men than for women. Despite of gender, a decrease in sexual desire during quarantine was found compared to before the lockdown, but for men this reduction did not reach statistical significance. A general decrease in sexual behaviors during lockdown was found. Among those who continue to engage in sexual activity, 25.66% reported a higher frequency in solo masturbation, 20.18% reported having more sexual intercourse with their partners, and 19.47% reported watching more porn. Those who had a decrease in frequency of sexual behaviors, reported a reduction in sexual intercourse with their partners (33.27%), a lower frequency in solo masturbation (24.96%), and a reduction in watching pornography alone (21.59%). Moreover, responders in a serious relationship were more likely to report an increase in sexual activity compared to those who were single or dating casually (Wignall et al., 2021).

However, men and LGBT respondents (around one-fifth of the sample), compared to women and heterosexual people, were more likely to report an increase in several sexual behaviors. Moreover, this trend was greater for those who were in a relationship with respect to those who were single or dating casually (Wignall et al., 2021).

Overall, during quarantine sexual desire and sociosexuality scores did not predict general health or perceived well-being. Nonetheless, a high desire to engage in casual sex was associated with a greater negative impact on subjective well-being for women, but not for men (Wignall et al., 2021).

On one hand, high-anxiety situations, such as the COVID-19 pandemic, seem to negatively affect relationship functioning and sexual activity, on the other hand, intimate and sexual connection with a partner can mitigate stress in such conditions (Mollaioli et al., 2021). Mollaioli et al. (2021) investigated the impact of containment measures of COVID-19 on the psychological, relational, and sexual well-being in a sample of 2,401 Italian people. The online survey, administered from April 7 to May 4, 2020, revealed that most of the participants (61.8%) reported no sexual activity vs. a smaller number (38.2%) who were sexually active—the majority were married or cohabitants. Interestingly, the latter showed lower-level anxiety and depression and a higher level of dyadic cohesion and satisfaction than the former. The interruption of sexual activity seemed to be due to quarantine-associated distress and impossibility to reach the partner. Interestingly, results showed that the frequency of sexual activities has an impact on psychological well-being, dyadic relationship, and sexual function/disfunction. Authors found that people who reported fewer sexual activities had more anxiety and depression-related symptoms and more sexual disfunction. On the contrary, participants who reported more sexual activities had better sexual functioning and a better dyadic adjustment. In both genders, sexual activity played a protective role on the development of anxiety and depression symptoms due to quarantine (Mollaioli et al., 2021).

Panzeri et al. (2020) found that certain variables were more often associated with pleasure, satisfaction, desire, and arousal levels in cohabiting persons. Through an online survey in Italy conducted from April 11 to May 4, 2020 (the 5th and 8th weeks after the start of lockdown), Panzeri et al. (2020) found that most of the cohabiting couples who took part in the study did not perceive any differences in their sexual life. In relation to sexual desire, men (69.7%) more than women (54.9%) said that they did not notice any differences with respect to the pre-pandemic period. The same pattern was found about arousal, with 72.7% of men and 58.2% of women reporting unchanged levels of arousal. A minor percentage of the sample felt an increase in sexual desire (12.1% of men and 18.7% of women) and arousal (15.2% of men and 20.9% of women) during lockdown. Gender differences were also found about orgasms: a higher number of women (17.6%) had trouble in reaching orgasm compared to men (6.1%). Moreover, men (15.2%) more than women (3.3%) reported that they reached orgasm faster and more frequently. Moreover, Panzeri et al. (2020) found that aging and anxiety played a role in the decrease in the perception of the quality of the marriage and of couples' satisfaction. Older participants reported fewer moments of intimacy with their partner caused by spending all the time with family and offspring and facing their daily life without the help of babysitters and parents. Furthermore, high levels of anxiety due to the fear of infection, for themselves and for their children, and COVID-related issues may have negatively affected their relationship satisfaction (Panzeri et al., 2020).

On one hand, increase in sexual desire, arousal, orgasm, and frequency of sex was associated with having more free time, spending more time with a partner, and feeling less stressed and more bored. On the other hand, respondents who felt a decrease in sexual desire, arousal, orgasm, and frequency of sex said that the reason for these changes was due to stress, forced cohabitation, routing, anxiety and worry about the job and the situation, feeling partner distance, and lack of privacy (Panzeri et al., 2020).

Despite that overall changes in sexual life during the lockdown were similarly reported between male and female participants, the latter reported more negative effects on the quality of their sexual life. People who reported a higher number of changes in sexual life during the pandemic seemed to experience more anxiety, depression, and stress (Panzeri et al., 2020).

Costantini et al. (2021) found that improvement in sexual lives during lockdown was associated with spending time, sharing interests, and being happy and satisfied with their partner. In their study, conducted from May 4 to May 18, 2020, on an Italian sample, authors investigated the changes in sexual behavior of individuals in a stable relationship for at least 6 months, who were sexually active and aged over 18 (Costantini et al., 2021). Almost the half of responders (49%) reported an improvement in the couple's sex life during Italian Phase 1 of lockdown, while the remainder of participants reported a deterioration or no changes, 29 and 22% of respondents, respectively.

Those who found an improvement in the couple's sex life were mostly women, with a long-lasting relationship, cohabiting with their partner, married, and/or without sons. Despite gender, those who felt an improvement in their sex lives had a good relationship with their partner: they shared all or some interests with their partner, liked the same activities as their partner in their free time, were happy and satisfied with their partners, and would remarry the same person. The improvement felt by those who lived with partners, in a long-lasting relationship, were probably due to the increased time spent together, sharing recreational activities and daily routines which previously could not be shared due to the lack of time. These results are in line with previous findings revealing that positive romantic relationships are associated with physical and mental health (Pietromonaco and Collins, 2017). Moreover, residents in central or south Italy, who had graduated, were aged over 40, and/or working at their usual workplace were more likely to report an improvement in the couple's sexual lives (Costantini et al., 2021).

On the contrary, those who felt that their sexual lives were worsening were mostly not living with their partners. Among those who lived with their partner, the worsening of the couple's sexual life was associated with having kids and having a stable relationship. Half of those who reported a decline of their sexual lives were living in the northern regions of Italy. Moreover, the worsening of sexual lives was more likely to be reported by those who attended high school and those who worked in smart working (Costantini et al., 2021).

Differences in gender were found in those who reported a worsened couple's sex life. Women who reported a worsened couple's sex life were more likely to have emotional difficulties such as anxiety, tension, fear, and insomnia than those who reported no worsening; although no pathological Female Sexual Function Index (FSFI-19 items) score was found. Moreover, women living with their partner who reported a worsened couple's sex life were more likely to be dissatisfied with and/or nervous of their partner. On the other hand, men who reported a worsened couple's sex life reported mild erectile dysfunction, orgasmic dysfunction, and a decrease in sexual satisfaction. In addition, men living with their partner who reported a deterioration of their sexual lives said they felt more uncomfortable with their spouse and had anxiety symptoms. A small percentage of men reported that they were unhappy with their partner and that they would have married another person. Among men living without their partners, the reduction of the International Index of Erectile Function (IIEF-15 item) scores was associated with a deterioration of couple's sex lives, except for sexual desire and symptoms of anxiety. In both genders, the worsening in sex lives was associated with being unemployed or working remotely, living in a northern region of Italy, having sons, and being in a relationship for more than 5 years (Costantini et al., 2021).

Finally, respondents who reported no changes were for the most part men, with shorter relationships, or without sons (Costantini et al., 2021).

During the COVID-19 outbreak there was an increase in sexual dysfunctions, nonetheless, spending more time with a loved one did help with negative pandemic-related outcomes (Karagöz et al., 2020). A cross-sectional study conducted from May 6 to May 20, 2020, investigated the changes in the sexual lives of couples, married or cohabitants, in Turkey (Karagöz et al., 2020). During the COVID-19 outbreak, 75.7% of male respondents and 76.3% of female respondents reported spending more time with their partners and 21.8% of men and 32% of women perceived restrictions during the pandemic to be positive in their daily and emotional relationship. Findings showed higher levels of anxiety and depression in women compared to men. Women more than men avoided sexual closeness with their partner because of concerns about spreading COVID-19, were more likely to think they could be infected during sexual intercourse, and took precautions like using condoms. Moreover, during the epidemic there was an increase in solitary sexual activity (i.e., masturbation, pornography consumption, etc.) which was significantly higher in male than in female participants. Gender differences can be partially explained by the fact that men are more likely to engage in solitary or dyadic sexual activity (Baumeister et al., 2005) and that sexuality is still taboo in Turkey (Karagöz et al., 2020). Nonetheless, responders of both sexes reported a significant decrease in the frequency of sexual intercourse during the pandemic compared to the pre-pandemic period (Karagöz et al., 2020).

For men, IIEF-15 scores revealed a significant decrease in erectile and orgasm function and sexual satisfaction during the pandemic compared to before the outbreak. Erection function was negatively associated with economic loss and anxiety and depression symptoms measured by Generalized Anxiety Disorders-7 (GAD-7), Patient Health Questionnaire-9 (PHQ-9), and Perceived Stress Scale (PSS). For women, results showed that a decrease of the total FSFI score was found to be negatively correlated with age and anxiety and depression symptoms. Moreover, a statistically significant reduction was found just in the domains of lubrification, orgasm, and satisfaction (Karagöz et al., 2020). These findings are in line with previous research stating that the etiology of orgasmic and ejaculatory disfunctions includes stress, anxiety, and concerns (Gomes and Nobre, 2012; Cohen and Goldstein, 2016). Finally, higher scores in all IIEF-15 domains and in total FSFI and other domain scores were shown to be more likely in people who reported spending more time with their partners, compared with those who did not (Karagöz et al., 2020).

### Using Sex as a Coping Strategy

The COVID-19 outbreak is affecting the sexuality of single people or those in a relationship in different ways. Despite of the pandemic's impact, some people are adapting and reinventing their sexuality. For instance, in an online survey conducted from March 21 and April 14, 2020; Lehmiller et al. (2020) found that 20.3% of the sample reported one to four (or more) new additions to their sexual life such as trying new sexual positions, sharing and/or acting out sexual fantasies, sharing nude photos, sexting, having cybersex, watching porn, filming oneself masturbating, visiting “camming” sites, tracking sexual behavior, and using high sexual technologies as virtual reality porn. Younger, less financially well-off, people living alone, LGBTQ+, and/or racial minorities were more likely to try new additions. Moreover, living with a partner or living alone entailed the adoption of different types of new sexual activities: people living with a partner were more likely to try new sexual activities involving their partner like trying new sexual positions and acting out sexual fantasies than people living alone. On the opposite side, those who lived alone reported higher rates of virtually and technology-based behaviors, like sexting and sending nude photos. Despite of gender, results showed that those who reported more sexual desire in the past 2 weeks were more likely to try new activities. For men only, loneliness was associated with new sexual activities. For women only, stress, desire for sex with a partner, and loneliness were related with such activities. Based on these findings, the authors inferred that trying new activities could partly reflect a coping strategy to fight negative consequence resulting from social distancing and pandemic-related distress (Lehmiller et al., 2020).

Overall, only trying new sexual activities with a partner (trying new positions, acting out fantasies, engaging in BDSM, and giving massages), but not common technology-based additions, were related to improvements in sexual lives. It is plausible that the increase in the use of technology-based sexual activity is a temporary coping strategy that will be replaced in favor of in-person interaction (Lehmiller et al., 2020).

The literature revealed that a greater use of pornography is associated with coping and boredom (Carvalheira et al., 2015). Quarantine measures to prevent contagion triggered a boost of consumption of pornography (Zattoni et al., 2020; Pornhub Insights, n.d.). By collecting data from January 9 to May 25, 2020; Zattoni et al. (2020) found that *Pornhub* was the main porn website searched in the Google search bar during quarantine, especially in countries with greater restrictions, such as Italy. In some countries the access to this porn website was higher than the period preceding the quarantine; even before and after the possibility offered by Pornhub to get free access to a premium account. In other nations, an increase in the use of pornography was noted although Pornhub did not give the opportunity to enjoy the free premium account. Moreover, the pandemic influenced not only the frequency of people watching porn, but even the emerging of a new kind of porn: coronavirus-themed porn. It includes sex activities with masks, gloves, and hazmat suits. In many countries where sex education programs do not exist, pornography can also be interpreted as a “tool” of sexual education (Eleuteri and Terzitta, 2021); during the COVID-19 emergency, videos of sexuality with medical masks could create confusion above “how to make love” following the previous outlined guidelines.

Alongside the boost of consumption of pornography, an increase in sexting was found during the pandemic, that is an increase in sharing messages, photos, and videos with sexual content (Chalfen, 2009; Lehmiller et al., 2020).

Bianchi et al. (2021) investigated the relationship between COVID-related stress, coping strategies, and three forms of sexting: experimental, risky, and emotional. Sexting is experimental when sexual contents are shared with a trusted partner (Wolak et al., 2012; Eleuteri et al., 2017); risky—related with other dysfunctional behaviors like substance use—when sexual contents are shared with strangers (Morelli et al., 2021); and emotional, when sexting is aimed at reducing negative feelings (Bianchi et al., 2019). An online study was conducted from April to May, 2020 on a sample of 1929 mostly Italian (96.7%) young adults aged from 18 to 29 years old. Results showed that among those who were in a long-distance relationship during quarantine (42.4%), the frequency of experimental sexting was greater compared to those who were in a non-distance relationship or single. This finding can indicate an attempt to maintain intimacy with their partners. Those who were single (40.4%) reported a greater frequency of risky sexting behaviors, compared to the others. A total of 16.7% of respondents, who were in a non-distance relationship during quarantine, reported the lowest frequency of emotional sexting behaviors, probably because of the emotional support received from their partners (Bianchi et al., 2021).

Authors found that experimental sexting behaviors were used to promote emotional closeness with their partner, rather than to escape from their negative feelings. The experimental form of sexting was not predicted by COVID-related stress while it was predicted by an adaptive coping strategy (i.e., social support) and lower tendency to find emotional support in religious practices. It also found an indirect relation between COVID-related stress and experimental sexting mediated by social support, so that, it seems that engaging in experimental sexting behaviors could represent an adaptive coping strategy by which people during quarantine looked for affection and support in their relationship in order to face COVID-19 related stress (Bianchi et al., 2021).

On the other hand, a direct association between risky sexting and COVID-related stress was found, as well as dysfunctional coping strategies. Pandemic-related stress was positively associated with avoidance coping and negatively associated with problem solving. In such a stressful situation like the pandemic, people, especially those with poor problem-solving abilities, can adopt a more dysfunctional coping strategy (i.e., emotion-oriented) rather than problem-oriented (Bianchi et al., 2021).

Finally, it was found that COVID-related stress, not turning to religion, and adaptive and maladaptive coping strategies were predictors of emotional sexting. Authors suggested that emotional sexting can be adaptive, if it is the result of looking for social support to face COVID-related stress, or maladaptive, if it is the outcome of avoidance strategies to cope with pandemic-related stress or when it is adopted when no appropriate problem-oriented solutions are available (Bianchi et al., 2021).

In a survey conducted from April 19 to April 21, 2020; Gillespie et al. (2021) investigated the use of sex to face negative feelings due to quarantine-related social distancing, loneliness, and difficulties in emotion regulation. Participants were female (66.2%), male (32.6%), non-binary (0.6%), and not specified (0.6%), residents of the United Kingdom, and aged 18–60. Compared to the 2-week period right before lockdown, during quarantine, participants did not report a significant difference in the average levels of coping using sex: on one hand, 30% of responders reported an increase of coping using sex during lockdown relative to immediately before that period while 29 and 41% of participants reported a decrease or no change in using sex to cope, respectively. Scores in the Coping Using Sex Inventory (CUSI) subscale assessing consensual coping using sex, revealed that being male, being younger, and experiencing more difficulties in emotion regulation were related with an increase in using sex to cope. When looking at sexual thoughts and behaviors with themes of rape and violence, participants who were male, younger, not complying with social distancing measures, and living alone were more likely to have endorsed at least one thought related to rape/violence. Differences in gender were found: in men, but not in women, loneliness was more likely to be related to the use of sex to cope, consensual or not; in women, but not in men, difficulties in emotion regulation were related to a greater likelihood of endorsing items with themes of rape/violence (Gillespie et al., 2021).

### LGBT People's Sexuality in the Pandemic

Particular attention should be given to COVID-19's impact on LGBT people's sexuality. Quarantine has had a negative impact on people's mental health, quality of life, and well-being, triggering anxiety, depressive and post-traumatic symptoms, and sleep disorders (Cao et al., 2020; Cellini et al., 2020; Gawai et al., 2020; Li S. et al., 2020; Pera, 2020; Salari et al., 2020). These outcomes may be more striking in lesbian, gay, bisexual, and transgender (LGBT) individuals. During COVID-19, LGBT people seems to have been more at risk of social and physical isolation and their condition was worsened by living with families who did not accept them and being away from friends and other relevant sources of support (Brennan et al., 2020; Barrientos et al., 2021). These distressors may have had an impact on LGBT people's sexuality.

In Shevlin et al.'s (2020) online survey, COVID-19 impacts on general well-being, sexual and substance use behavior, and HIV and sexually transmitted infection prevention and treatment in US MSM were examined. A large proportion of participants reported a decrease in quality of life (69.4%), quality of sleep (37.7), connection with friends (56.1%), and with family (28.9%). A smaller percentage reported problems related to buying food, paying rent, decreasing work hours or losing their job, and a greater need to support loved ones who lost their job. Moreover, 72.7% of participants reported increased anxiety levels (Sanchez et al., 2020).

For sexuality, 48% of respondents reported no changes in the number of sexual partners, 51.3% of respondents reported a decrease in the number of sexual partners, and only 0.9% of respondents reported an increase. Although social distancing measures reduced in-person interactions and flirting for MSM, dating applications became a key means of connection. Indeed, a percentage of respondents reported an increase or no change in the use of dating or hook-up apps to meet (5.8 and 44.9%, respectively), or connect (14.9 and 49.7%, respectively), with other men. Almost all the sample reported that condom access and usage were unchanged, although a high number of respondents reported increased difficulties to get access to HIV or STI testing and STI treatment. Finally, findings showed that 10 and 25% of participants had increased the use of recreational drugs and alcohol, respectively (Sanchez et al., 2020).

Overall, younger participants (15–24 years) were more likely to report an impact of COVID-19 on economic resources, an increase in the use of apps to connect with other men, more problems accessing condoms and HIV and STI testing and treatment services, and an increase in alcohol and drug use.

Further findings showed a moderate increase in the number of sexual partners among MSM and an increase in casual sex (Shilo and Mor, 2020a; Stephenson et al., 2020).

Stephenson et al. (2020) investigated changes in sexual behaviors in a sample of 518 gay, bisexual, and other men who have sex with men (GBMSM). This sample consisted of people assigned male sex at birth, aged between 18 and 44, currently residing in the US and in its dependent areas, and reporting sexual experiences in the past 12 months. Authors assessed respondents' experiences of COVID-19 (i.e., employment status, housing stability, food security, use of drugs, and use of or binge drinking) and perceptions of the prevalence of COVID-19. Moreover, changes in sexual behavior since the COVID-19 epidemic started (from February 2020 to April/May 2020) compared to 3 months prior to the pandemic (from November 2019 to January 2020), and HIV prevention behaviors were investigated. Results showed only 66.9% of the sample believed that COVID-19 could be contracted via sexual activities. While 94.8% of respondents believed that contagion could happen through kissing, roughly half of the participants believed it could be transmitted through other sex acts (i.e., oral sex, anal sex, rimming). Men reported that it was not important to reduce the number of sexual partners during COVID-19 and showed willingness to continue their sexual activity during the pandemic. Compared to the previous 3 months, respondents reported a moderate increase in sexual partners and a mild increase in the number of unprotected anal sex partners (Stephenson et al., 2020). Despite there being no evidence that the virus can be transmitted via semen (Guo et al., 2021), contagious contact is very likely in in-person sexual activities because it requires people to be in close physical contact with others (World Health Organization, 2020). In this vein, it is possible that men are just looking at the perceived lower likelihood of transmission through sex, underestimating the threat in close physical contact. Furthermore, substance use increase was related to a greater likelihood to have protected and unprotected sexual intercourse (Stephenson et al., 2020): previous research showed that drug use contributes to risky sexual behavior (Woody et al., 1999). Having more free time during the quarantine and daily routine disruption may have led to more time spent in sexual activities and substance use. Moreover, it is possible that both sexual activity and substance use increase may represent coping strategies to face quarantine-related distress (Dariotis and Chen, 2020; Gillespie et al., 2021). Finally, higher perceived levels of COVID-19 at the country and state level were associated with a lower increase in the number of sexual partners, while higher perceived levels of COVID-19 among their sexual partners were associated with a lower increase in the number of unprotected anal sex partners (Stephenson et al., 2020).

In a similar vein, an online survey conducted on Israel MSM (Shilo and Mor, 2020a) found that out of a total of 1,012 participants, 39.5% did not adhere to COVID-19 restrictions and had casual sexual intercourse. Among these, 84% had had more than 3 sexual partners, 2.1% had had more than 10 sexual partners, and 2.4% took part in in-house orgies. With respect with those who adhered to COVID-19 distance restrictions, men who had casual sex were more likely to be single, young, distressed, and less educated. Exploring sexual behaviors before and during the restrictions period, results showed that 72.1% of those who had causal sex had a decrease in casual partners and sexual risky behaviors (kissing, having anal and oral sex, using alcohol or drugs during sex, paying or being paid for sex) than before with respect of those who did not. Furthermore, compared to those who used to have casual sex before the pandemic but stopped after the COVID-19 outbreak, MSM who continued to engage in casual sex were more likely to engage in sexual behaviors (having anal sex, using sex toys during intercourse, paying or being paid for sex) before the pandemic.

For sexual desire, 23.1% of participants reported a reduction in desire during the restrictions period than in the pre-pandemic period (Shilo and Mor, 2020a).

Moreover, during the social-distancing period, respondents who had casual sex were more likely to use condoms or Pre-Exposure Prophylaxis (PrEP) than before the pandemic (Shilo and Mor, 2020a).

The means to meet other men were mainly dating applications and social media. Almost the entire sample (90%) used dating applications during the social-distancing period, but only 37.7% reported an increase in the use of these resources during the social-distancing period than before the outbreak (Shilo and Mor, 2020a).

Overall, the increase in the use of phones or webcams and pornography during the restrictions period, compared to before the pandemic, was significantly associated with higher levels of mental distress (Shilo and Mor, 2020a). Pornography consumption can be seen as a coping mechanism to face stress due to uncertainty and insecurity (Mestre-Bach et al., 2020; Velotti et al., 2020; Zattoni et al., 2020). Nonetheless, pornography consumption or using remote devices for sexual satisfaction were not a predictor of abstaining from casual sex during the pandemic. Moreover, 40% of respondents reported having used dating applications and social media to find sexual partners (Shilo and Mor, 2020a).

Finally, 3.2% of respondents showed willingness to have sex with someone who had COVID-19 and 30.1% of respondents showed willingness to have sex with someone diagnosed with HIV. Worse life condition and health outcomes are associated with HIV compared to COVID-19. Nonetheless, the lack of effective vaccine and medical treatment and the distressing news make people feel more threatened by having sex with a partner affected by COVID-19 than HIV (Shilo and Mor, 2020a).

Engaging in casual sex during the social restrictions period due to the pandemic was found to be associated with being younger, being single, engaging in riskier sexual behaviors before the COVID-19 outbreak, and showing lower levels of well-being and high levels of mental distress (Shilo and Mor, 2020a).

## Discussion

The state of health emergency due to the COVID-19 pandemic and the measures adopted to reduce its spread have had a strong impact on health, economies, and societies worldwide. Post-traumatic stress, anxiety, depression, sleep disorders, rejection, fear, guilt, pain, loss, confusion, and anger are found to be the most common psychological responses to the pandemic (Brooks et al., 2020; Chew et al., 2020; Gawai et al., 2020; Ibarra et al., 2020; Pera 2020). All these outcomes have been raised from the long-lasting isolation, the fear of contagion, the frustration, the boredom, the lack of information, the financial loss, and the stigma. Furthermore, the similarity of COVID-19 symptoms with those of other diseases, the concern for children, the fear of losing loved ones, and the lack of support to vulnerable individuals are other threats to psychological well-being (Brooks et al., 2020). The COVID-19 pandemic has also had a strong impact on individuals' and couples' sexuality (Eleuteri and Terzitta, 2021). The primary aim of this review was to investigate how the COVID-19 outbreak and its consequences impacted people's sexuality.

Overall, a certain reduction in sex frequency was found, mainly in singles and in those that were unable to reach their partners (Karagöz et al., 2020; Li W. et al., 2020; Luetke et al., 2020; Schiavi et al., 2020; Coombe et al., 2021; Mollaioli et al., 2021; Wignall et al., 2021).

For healthcare workers, the increase in workload and higher risk contagion, for themselves and for their loved ones, due to close contact with COVID-19 patients, negatively influenced their emotional and sexual sphere (De Rose et al., 2021).

Previous research found that women are more likely to be affected by the COVID-19 pandemic exhibiting psychological issues such as stress, anxiety, depression, and post-traumatic stress symptomatology (Liu et al., 2020; Wang et al., 2020). In addition to specific couple issues, psychological difficulties and emotions during the COVID-19 lockdown played a role on people's sexuality. This is in line with previous studies that found a positive correlation between stress, anxiety and depression, and the negative impact on sexuality, especially in women (Lykins et al., 2006; Kalmbach et al., 2014; Cocci et al., 2020; Karagöz et al., 2020; Vizard et al., 2020). Women's sexuality was found to be more at risk of pandemic detrimental effects compared to men (Cocci et al., 2020; Karagöz et al., 2020; Schiavi et al., 2020; Yuksel and Ozgor, 2020; De Rose et al., 2021; Wignall et al., 2021). Women working outside the home during quarantine and those with a higher level of education were more likely to experience a significant worsening in sexual function and quality of life, a reduction in the mean number of sexual intercourse sessions per month, and a higher level of distress (Schiavi et al., 2020). Moreover, in women, but not in men, a high desire to engage in casual sex was associated with a greater negative impact on subjective well-being (Wignall et al., 2021).

On the other hand, men who reported a worsening in the couple's sex lives during the pandemic also experienced mild erectile dysfunction, orgasmic dysfunction, and a decrease in sexual satisfaction (Karagöz et al., 2020; Costantini et al., 2021). Erection function was negatively associated with economic loss and anxiety and depression symptoms (Karagöz et al., 2020). Men living with their partner who reported a deterioration of their sexual life were found to be unhappy with their partners and to have anxiety symptoms. Among men living without their partners, the IIEF-15 item score was associated with a deterioration of the couple's sex lives, except for sexual desire and symptoms of anxiety (Costantini et al., 2021).

However, men, especially if in a relationship, compared to women, were more likely to report an increase in several sexual behaviors (Karagöz et al., 2020; Wignall et al., 2021).

Gender differences can be due to the increase of stress related to additional domestic labors. In addition, school closures and the cessation of extracurricular activity may have decreased the possibility to have intimate moments with partners. Indeed, having one or more children was found to be associated with a reduction in FSFI scores during the pandemic (Schiavi et al., 2020). Moreover, these findings can be partially explained by the greater engagement of men in solitary or dyadic sexual activities, compared to women (Baumeister et al., 2005) and by the fact that sexuality is still taboo in some countries (Karagöz et al., 2020).

Among couples, COVID-related stressors may predict a decrease in relational satisfaction and an increase in maladaptive relationship behaviors, such as conflict (Balzarini et al., 2020; Luetke et al., 2020). The disruptions of routine for individuals and families have contributed to increases in stress and exacerbation of conflict between partners. Decreases in solo and partnered sexual activity were found to be associated with an increase in COVID-19-related conflicts. Moreover, conflict was associated, in a non-significant way, with lower levels of self-reported orgasm and emotional connection with a partner (Luetke et al., 2020).

For age, during the quarantine compared to before, Wignall et al. (2021) found a decrease in sexual desire and a general decrease in sexual behaviors in a sample of young adults, aged 18–32. In another study, Cocci et al. (2020) found that sexual dissatisfaction in both men and women was significantly correlated with younger age and depression levels. The literature showed that young adults cope with many normative transitions (Arnett, 2000) including educational and professional development, social and romantic relationships, and living away from family. These changes may represent a stressful situation which may have been worsened by the COVID-19 outbreak, influencing the mental and sexual health of younger people (Shanahan et al., 2020).

On the other hand, Panzeri et al. (2020) found that aging played a role in decreasing perceived quality of marriage and couples' satisfaction. Older participants reported fewer moments of intimacy with their partner caused by spending all the time with family and offspring and facing their daily life without the help of nannies and parents.

Overall, worsening of sexual life seems to be related to conflict, emotions and psychological difficulties, being female, being single or away from a partner, being a healthcare worker, and having children (Cocci et al., 2020; Luetke et al., 2020; Schiavi et al., 2020; Shevlin et al., 2020; Coombe et al., 2021; Costantini et al., 2021; De Rose et al., 2021). Moreover, stress, forced cohabitation, routine, anxiety and worry about the job and the pandemic, feeling partner distance, being unhappy with their partner, and lack of privacy were associated with a decrease in sexual desire, arousal, orgasm, and frequency of sex (Panzeri et al., 2020; Costantini et al., 2021).

On the other side, during the pandemic, some people, mainly those living with their partners, found an increase in the frequency of sexual intercourse (Coombe et al., 2021; Wignall et al., 2021). Some others said they had engaged in group sex, swinging, or threesomes since lockdown began, compared with 2019 (Coombe et al., 2021). Panzeri et al. (2020) found that people who spent more time with their partners and felt less stress and more bored also experienced an increase in sexual desire, arousal, orgasm, and frequency of sex.

Mollaioli et al. (2021) found that those who had sex during lockdown showed a decrease in anxiety and depression levels and a higher level of dyadic cohesion and satisfaction, compared with those who did not. On the contrary, a reduction of the frequency of sexual activity was more common between those who reported higher levels of anxiety, depression, and stress and an increase in sexual disfunction (Panzeri et al., 2020; Mollaioli et al., 2021). In this vein, sexual activity seems to play a protective role on individual psychological health.

In general, improvements in sexuality were associated with living happily with a partner, being happy and satisfied with a partner, feeling less stressed and more bored, having more free time, having fewer recreation opportunities, and having minor workload (Arafat et al., 2020; Panzeri et al., 2020; Costantini et al., 2021).

For changes in sexual behaviors, an increase in the use of sex toys and masturbating was found during the pandemic, especially by people who reported less or no sex during the lockdown compared with those who reported more sex (Coombe et al., 2021). Moreover, during the pandemic, an increase in sexual experimentations was found. People who lived with their partners during the pandemic were more likely to try new sexual activities involving partners like new sexual positions and acting out sexual fantasies, than people who lived alone. On the opposite side, those who lived alone reported higher rates of virtually and technology-based behaviors (Lehmiller et al., 2020). Nonetheless, only trying new sexual activities with a partner, but not common technology-based additions, were related to improvements in sexual lives.

Quarantine measures to prevent contagion triggered a boost of consumption of pornography (Zattoni et al., 2020; Pornhub Insights, n.d.). The pandemic influenced not only the frequency of watching porn, but even the emergence of a new kind of porn: coronavirus-themed porn. It is possible that this new trend is the expression of the need for sexual novelty and the ability to fetishize virtually everything. The growing engagement in use of pornography could be due to boredom (Carvalheira et al., 2015; Zattoni et al., 2020). Moreover, in addition to having a recreational function, watching porn can represent a strategy to cope with negative emotions, stress, loneliness, depressive feelings, and fear of death (Shilo and Mor, 2020a; Velotti et al., 2020; Zattoni et al., 2020). In this vein, a recent study found an increase of coping using sex (Gillespie et al., 2021). When looking at sexual thoughts and behaviors with themes of rape and violence, participants who were male, younger, not complying with social distancing measures, and living alone were more likely to have endorsed at least one thought related to rape/violence (Gillespie et al., 2021).

The quarantine's negative impact on mental health (Cao et al., 2020; Cellini et al., 2020; Gawai et al., 2020; Li S. et al., 2020; Pera, 2020; Salari et al., 2020) may have been more striking in lesbian, gay, bisexual, and transgender (LGBT) individuals. Previous research showed that, compared to heterosexual people, LGBT people are more likely to suffer from psychiatric disease such as anxiety and depression disorders (Cochran and Mays, 2000) than heterosexual people (King et al., 2008). Cochran et al. (2000) found that lesbians reported using alcohol more frequently and in greater amounts than heterosexual women. Moreover, homosexual and bisexual men are at greater risk of suicide and depressive symptoms than heterosexual men (Cochran and Mays, 2000). Furthermore, compared to heterosexual men, MSM are more likely to be single (Shilo and Mor, 2020b) and this may lead to an increase in mental distress and perceived loneliness (Shilo and Mor, 2020a).

During COVID-19, LGBT people seems to have been more at risk of social and physical isolation and their condition was worsened by living with families who did not accept them and being away from friends and other relevant sources of support (Brennan et al., 2020; Barrientos et al., 2021). These distressors had an impact on LGBT people's sexuality (Sanchez et al., 2020).

Because of social distancing measures, dating applications have become a key means of connection, especially for younger MSM (Sanchez et al., 2020; Shilo and Mor, 2020a). Despite of COVID-related restrictions, an increase, or no change, in the use of dating or hook-up apps to meet or connect with other men was found. Moreover, GBMSM reported no changes or an increase in the number of casual sexual partners (Sanchez et al., 2020; Shilo and Mor, 2020a; Stephenson et al., 2020). It is possible that men are just looking at the perceived lower likelihood of transmission through sex, underestimating the threat of being in close physical contact. Furthermore, the lack of effective vaccine and medical treatment and the distressing news make people feel more threatened by having sex with a partner affected by COVID-19 than HIV (Shilo and Mor, 2020a).

Substance use increase was related to a greater likelihood to have protected and unprotected sexual intercourse. Having more free time during the quarantine and daily routine disruption may have led to more time being spent in sexual activities and substance use. Moreover, it is possible that the increase in sexual activity and in substance use may represent coping strategies to face quarantine-related distress (Dariotis and Chen, 2020; Gillespie et al., 2021).

Engaging in casual sex during the social restrictions period due to pandemic was found to be associated with being younger, being single, engaging in riskier sexual behaviors before the COVID-19 outbreak, and showing lower levels of well-being and high levels of mental distress (Shilo and Mor, 2020a).

The lack of support and intimacy with family and friends due to COVID-19 restrictions may have worsened vulnerability to depression and negative mental outcomes of LGBT people. The sense of loneliness is typically associated with low levels of welfare and labeled as a risk factor for mental disease (i.e., depression, anxiety, stress, insomnia; Banerjee and Rai, 2020). In this vein, it is possible to speculate that single MSM, who felt more alone and more emotionally distressed, were more likely to break restriction rules and engage in casual sex (Shilo and Mor, 2020a).

The state-of-the-art literature represents the first attempt to understand the changes in couples' and individuals' sexuality, during a stressful situation such as the COVID-19 pandemic. Among the strengths we find the investigation of certain factors (i.e., gender, employment, parenthood, relational status, etc.) are related with the changes in the sexual sphere and of the transformation of people's sexuality to face COVID-related distress, paying attention to different gender and sexual orientations.

Despite of these strengths, the current literature has some limitations. First, most of the research was conducted online because of pandemic-related restrictions. Thus, most of respondents were good at using computers. Future research should investigate how the pandemic has affected the population that has not been reached by online surveys. Second, most of the samples examined were small and not homogeneous. It is likely that the current results are not adequate to capture the impact of COVID-19 on the sexuality of different population groups that vary, for example, for age, culture, language, socioeconomic status, etc. Third, the large use of self-report data may be subject to social desirability, recall, and/or other biases. Moreover, in some societies, sexuality is still taboo making it difficult to find people who want to talk openly about their sexual lives. Fourth, as far as we know, most of the research was carried out in the early stages of the pandemic. Therefore, further studies are needed to investigate the short-term and long-term impacts that COVID-19 has had on our sexual lives. Fifth, most of the studies did not consider the emotional relationship and affectivity within couples, which are known to have an influence on couple's sexuality. Moreover, one's sexual behavior can be affected by partner sexual attitude, which could be a possible future research area. Future investigation should better explore the relation between media communication and sexuality. Finally, the samples are mainly composed of heterosexual, lesbian, gay, and bisexual people, leaving aside a slice of population (i.e., transgender, intersex, queer/questioning, asexual, non-binary, pansexual). Further studies are needed to understand how the COVID-19 pandemic is affecting the sexual lives of LGBT+ people and if the epidemic is affecting LGBT+ individuals differently than heterosexual, cisgender people.

## Conclusion

What emerged from this narrative review should be considered at a clinical level. The role of the emotional and relational problems on sexuality highlights the need to work on individuals' emotion, promoting their resilience, and reducing the spillover effect of stress into the relationship. Clinicians also might promote couples' cohesion and intimacy, that seem to have a protective role on the development of psychological disease during a stressful situation such as a pandemic. Moreover, encouraging people to trying new sexual activities with their partner can be useful to cope with a stressful situation and to improve the couple's sexual life. Special attention should be paid to categories mostly at risk of negative outcomes such as women and LGBT+ people, as well as individuals at risk for maladaptive use of sexting or pornography. Interventions to these latter risks should be focused on raising awareness on individual's own coping responses and on the implementation of functional coping strategies.

Until the end of the state of emergency, further analysis and follow-ups are crucial to continue to monitor people's sexuality changes and their implications on individual health. Longitudinal studies are necessary to explore whether the observed changes do persist or do not and whether and what social and/or health interventions are needed to mitigate the negative pandemic effects. Moreover, insights into which factors are associated with sexual improvements might be helpful to enhance people's intimate lives and pursue safe leisure activities during a potential future emergency condition.

## Author Contributions

SE and FA contributed to the conception and design of the work, drafting the article while FP and VS revised it critically for important intellectual content. All the authors provided approval for publication of the content.

## Conflict of Interest

The authors declare that the research was conducted in the absence of any commercial or financial relationships that could be construed as a potential conflict of interest. The reviewer LP declared a shared affiliation, with no collaboration, with one of the authors SE to the handling editor at the time of the review.

## Publisher's Note

All claims expressed in this article are solely those of the authors and do not necessarily represent those of their affiliated organizations, or those of the publisher, the editors and the reviewers. Any product that may be evaluated in this article, or claim that may be made by its manufacturer, is not guaranteed or endorsed by the publisher.
